# Treatment of Dental Caries When Complicated with an Irritable or Exposed Pulp

**Published:** 1856-01

**Authors:** James Taylor


### ARTICLE XI.

### *Treatment of Dental Caries when Complicated with an Irritable or Exposed Pulp.* By James Taylor.

Perhaps there is no department of surgical or medical science, which is demanding more attention at this time than that which heads this article.

We do not believe that in all the range of medical science, any one disease is receiving more careful study and enlisting more talent than this. Master minds and skillful surgeons are testing the utmost powers of our art to wrest from disease, organs heretofore considered hopelessly lost, however great may be the achievements of mechanical dentistry, and important the improvements here made, yet they cannot surpass the progress which is evidently being made in this particular department of dental science. The object of one is merely to repair the loss made by disease: the other to prevent that loss, and just so far as the natural organs are superior to artificial ones, is this subject paramount in importance to the other.

The skillful dentist who has kept pace with the improvements of the profession must feel some gratification in view of the fact; that, while many are disposed to sacrifice the *troublesome natural* organs so as to give place to the beautiful artificial, yet there are many equally disposed to combat the ravages of disease and restore these troublesome members to beauty and usefulness. Just how far the conscientious dentist may sacrifice utility to beauty is a matter which must be decided by the circumstances of the case, yet we think some consciences need education in this affair as well as other things.

A very interesting presentation of the subject we have chosen for this article would be to present the views of the profession on the treatment of exposed pulp, as now advocated, and ten to fifteen years since. This could be done by reference to articles then and now published—the contrast would likely be greater than most are aware of.

But we design not taking this view of the subject, but shall as we think, present a few practical thoughts on the anatomical and pathological relations which exist between the teeth themselves and the surrounding tissues which are more or less affected by the disease under consideration. As it regards the treatment, the entire subject will necessarily embrace—first, the symptoms and treatment of an irritable pulp before an utter exposure by decay—second, the symptoms and treatment of an exposed pulp with an object to its preservation, and third, the different means of destroying the pulp, and the practicability of preserving the tooth after this source of internal vitality has been removed.

Anatomically and pathologically studied, the teeth with their surrounding investments would seem to point out a beneficent structural organization and adaptation, whereby science might expect to meet and remedy many of the evil consequences of disease. We take it for granted here, and believe that the tooth is the best possible organization which a beneficent Creator could devise to subserve the purpose for which they are designed. Infinite Wisdom has certainly been displayed in their structure—how important the enamel, how skillfully and admirably its columns are arranged for strength and use. Where is the organization which could take its place, and alike resist the action of foreign substance which must necessarily be daily brought in contact with it? In elements it more nearly harmonizes with the dentine which it covers than any other substances which possesses its density and durability. The dentine, how very nearly it approaches the bones of the body, and yet for adaptation to the flint-like structure of the enamel how much more hard and firm is its structure, and of what use would be the enamel with a less solid base to rest upon? But how shall so dense a structure be adapted and made to harmonize with its more soft connections. To meet this condition of things, commencing at the edge of the gum or enamel and thickening to the apex of the fang is a covering of crusta petrosa or cementum, through which it is prepared for its connection with the periosteum, could anything be more admirably arranged? Well we would say, that this is mechanically most wisely constructed. Let us see if it is not anatomically so too. In the osseous structure the density is not such as to prevent an amount of circulation sufficient for its reproduction and maintaining of its vital connection with the soft part. But this would be insufficient for the organs of mastication, for a greater degree of strength and solidity is necessary for those organs which must first lay hold of, and prepare the food for the purposes of the animal economy. This very condition of things forbid a like circulation as is maintained through the bones of the body, but to meet this condition of things and maintain a vital connection, which shall subserve the requirements of the economy, we have first the usual periostial covering which attaches itself to the cementum—supplying it with a circulation and vitality, but which so feebly transmits its vital fluid through the dentine that it may be entirely suspended in this, without disarranging that maintained with the cementum. In this arrangement we see the wise provision which is made for an injury, accidental or from disease, which foresight must have anticipated for organs so much exposed.

The sources of internal vitality penetrate the tooth through the apex of the fang, and after having elaborated the organ, appear content to sustain through its dentinal portion, a sufficient degree of vitality to subserve the purposes of a living organization, and feebly protect itself in its bony canal from the encroachment of disease. This power of self-preservation is an important one, and we are satisfied when judiciously attended to in deep seated caries, many teeth can be easily saved as living organs, which are now, if saved at all, saved as dead ones.

The pulp appears designed to maintain the vitality of the dentine, and in this, sends through its tubular or cellular structure a coloring fluid which marks it as a living body. Every practitioner is soon enabled to tell by the hue of the organ whether the pulp is diseased or not. It is not fair to infer that the suspension of this circulation and the change which it undergoes, when retained in the tubuli, is the cause of the darkened hue which the teeth assume on the death of the pulp. Does the death of the pulp necessarily give us a dead organ throughout its entire structure? This is an important question, and on its proper solution, we should be governed in the treatment of these organs. If the death of the pulp only involves the loss of the vitality of the dentine, we may with propriety try to arrest the further progress of the disease. If, however, it involves loss of vitality in the cementum, the question arises: Will the periosteum maintain its connection with the dead osseous structure? What evidence have we that the alveolo-dental membrane supplies nourishment and vitality to the cementum, and does it ever do more than this? Does it, under favorable circumstances connect itself with the pulp organ through the dentine? To sustain the latter, we have the reported case of Prof. Mussey alluded to some year or two since in the Register, in which, on the removal of an inferior incisor, the labial portion of which, and also the point of the fang was laid bare by an absorption of the gum and alveolus, and yet the pulp was alive. The nerve and blood vessels which pass in and out at the apex of the root, had been severed by the disease for sometime, still, blood and a living nerve was found in its narrow canal. During the summer, having occasion to remove two such teeth for a patient, we split both for an examination, and found in one a living nerve and blood vessels apparently perfect.* It may also be remarked that nerves often remain living in teeth long after the crown is destroyed by disease. Still the question would be pertinent; are they kept so without any aid from the periosteum? It appears to us that the anatomical arrangement of the tooth with the enamel, dentine, cementum and periosteum, the first possessing little if any vitality, the second with increased sensibility, and the third, still increased in this particular, whilst the last, the periosteum has its usual amount of vital powers as displayed in other parts of the body, is specially arranged to provide for exigencies that may arise as the result of disease in organs so important. The vitality so gradually diminishes as we approach the enamel that no great disturbance to the vital functions takes place, if it is even cut short before it reaches this outer covering. For instance, the cementum may still maintain its healthy functions if the dentine be impaired. The dentine receiving its principal supply of vitality from the internal vessels, these may be removed and the tooth as connected with its alveolar investments, still be regarded as a living organ. We regard the cementum as an intermediary substance between the periosteum and dentine, and so peculiarly organized that the contact of the latter even in a dead state does not necessarily impair its proper function.

But if we take the position which the two cases of living pulps already referred to would warrant, we would suppose that

* We hope the profession will not let cases of a like character pass without an examination.

the periosteal vessels which supply the cementum penetrate also the dentine, and for a long time maintain a low degree of vitality in this. This, it is true, is a supposition, but let us see if the inference is not a fair one.

We are first met with the fact, that roots of teeth when their crowns have been removed, generally in a few years show signs of being regarded by the contiguous parts as foreign bodies. This although a general rule, is not universally so. There are numerous exceptions to this, and they often maintain their integrity so long that the cause demands investigation. Is it a peculiar idiosyncrasy of the system which adapts itself to this condition of things, or is it because the circumstances following the result of the disease which has occasioned the loss of the crown, have been such as would ordinarily result in a like preservation? We admit there are certain favorable, and also unfavorable conditions of the system—such as constitutional organization, etc., which would tend to a favorable or unfavorable issue as regards the preservation of healthy action, yet we feel as sure that there are certain circumstances generally involved in the loss of the crowns of the teeth by disease that are sufficient in and of themselves, to cause the surrounding parts to repel the roots as irritants. We must not forget that perfectly sound and healthy teeth even when invested in healthy alveolar processes and gums, when they have lost their antagonists, also soon show evidences of being regarded by the contiguous parts as no longer useful but unnecessary and foreign. Roots of teeth are relatively in the same condition, they have lost their antagonism. Roots of teeth to which artificial crowns have been attached sometimes last for twenty years, and why, because the antagonism is restored and we might add the dead and decomposed portions have been removed.

But we seek further cause why teeth often become irritants and act as foreign bodies, and we think we shall find it; not in the fact that the dentinal portion of the tooth has lost its vital principle from the death of the pulp, and hence acts as an irritant on the periosteum through the cementum; but the death of the pulp has made an impression on the periosteum at the apex of the fang where the pulp vessels pass through this membrane as they enter the teeth, and here begins that disease which so often separates the teeth from the surrounding parts, and this disease acts upon the membrane itself. We might adduce as evidence of this, the fact that the disease which manifests itself in repelling the roots, generally commences at the apex of the fang. The irritation is here first set up and the periosteum often is separated at this point, while around the main body of the root it maintains its integrity. We regard the decomposed pulp which is often left in the canal at the end of the root as a continued source of irritation to the periosteum, also all foreign matter subject to decomposition which may be forced from various causes into the nerve cavity. But we are anticipating our subject, for when we come to speak of the destruction of the pulp, and preservation of the teeth, we shall have occasion to elucidate this subject more fully. Having alluded somewhat freely to that anatomical organization of the teeth which is necessary to a proper understanding of the pathological changes which are developed in the progress of disease, we shall now consider the nature and treatment of deep seated caries resulting in irritation of the dental pulp before exposure.

Different authors describe a great many different varieties of this disease, more indeed than is necessary to a proper understanding thereof, a consideration at this time of the varied characteristics which the disease assumes would only cause confusion where we wish simplicity. For the sake of brevity and simplicity, we merely classify into three varieties, considering all others as mere modifications of these. The peculiar characteristics which accompany these forms of the disease should be understood and carefully observed in the treatment of deep seated caries. We use the words "deep seated caries," merely to express a condition of decay, and not a distinct form of disease. The disease must be deep seated when it has nearly reached the pulp. We classify as follows, first black caries, second brown, and then white. These forms of disease we regard as incidental to peculiar constitutional organization, habits of the individual, and proximate cause of disease.

A few words in relation to the particular character of these varieties of caries will be necessary to diagnose a proper treatment.

The black caries is what the name indicates. It is generally slow in its progress, rather hard of texture and possesses less sensibility than either of the others, there appears to be at no time, little if any inflammation of the dentine. The agent inducing the disease appears to act very feebly, scarcely possessing the power to disintegrate the constituents of the dentine; very often the agent having exhausted its powers, the disease becomes stationary. In this form of disease we but seldom find the pulp exposed. The irritation which it induces is ordinarily not more than sufficient to establish a deposition of bony matter by the pulp organ which it often does as rapidly as the disease advances—thus keeping up the relative distance between itself and its approaching enemy.

These are facts which should be borne in mind in our diagnosis of the proximity of the pulp, also the vis medicatrix naturæ to be relied on in arresting the disease.

The brown variety of caries progresses far more rapidly than the black, and just as far as it assimilates the latter, is its sensibility and progress diminished. In this form of the disease there is generally much sensibility of the dentine and often positive inflammation, layer after layer of the dentine is decomposed, the earthy portion being completely disintegrated, leaving the cartilaginous structure soft and flexible, very soon the disease becomes deep seated, either exposing the pulp or leaving a mere lamina of the dentine covering this organ in an inflamed condition, this lamina of the dentine being important as a covering to the pulp it is necessary if possible to restore it to health and preserve.

The white variety of caries is characterized by great sensibility, the rapidity of its progress and the removal of more of the cartilaginous structure than in the brown variety, sometimes the lamina of dentine under the disease is hard and dense and very sensitive, and then again less sensitive but penetrating deep and soon exposing the pulp. These particular forms of disease are, it is true, often much modified by the general constitutional health and the vitiated secretions of the mouth.—*Dent. Reg.*

(To be continued.)

